# Multimodal approach in distinguishing and managing uterine arteriovenous malformation: A case report

**DOI:** 10.1016/j.radcr.2024.10.075

**Published:** 2024-11-13

**Authors:** Sajeev Sridhar, Roman Sukhovershin, John A. Hancock

**Affiliations:** a Houston Methodist Research Institute, Department of Radiology, 6670 Bertner Ave, Houston, Texas, 77030, USA; b Houston Methodist Hospital, Department of Radiology, 6565 Fannin St, Houston, Texas, 77030, USA; c Houston Radiology Associated, 6565 Fannin St, Houston, Texas, 77030, USA

**Keywords:** Uterine arteriovenous malformation, CTA, MRI, Transarterial embolization, Transvaginal ultrasound, Doppler imaging

## Abstract

Arteriovenous malformations (AVMs) are abnormal vascular connections bypassing the capillary system, categorized as acquired or congenital. Acquired uterine AVMs, often resulting from uterine trauma due to procedures like dilatation and curettage, can be life threatening, necessitating prompt diagnosis and management. Here we present a 34-year-old woman with a history of missed abortion and dilatation and curettage presenting with abnormal uterine bleeding 2 months postprocedure. Although initial transvaginal ultrasound suggested retained products of conception, several modalities were required to accurately diagnose uterine AVM with invasive angiography revealing the culprit vessel. Multimodal imaging approaches are crucial for accurate diagnosis and treatment. This case highlights the importance of prompt and precise management to prevent severe outcomes and maintain fertility, emphasizing the need for continued research to improve treatment strategies.

## Introduction

Arteriovenous malformations (AVMs) are aberrant connections between arteries and veins that bypass the capillary system, involving tissues or organs. AVMs are generally categorized as congenital or acquired, with the latter being less common [1]. Recent years have seen a rise in reported cases of AVMs, a trend attributed to the increased frequency of invasive procedures [2]. Acquired uterine AVMs are predominantly observed in women of reproductive age, often resulting from uterine trauma associated with procedures such as dilatation and curettage (D&C), abortion, or operative delivery. Given the life-threatening potential of this condition, timely diagnosis and management are imperative. This case report discusses a 34-year-old woman who presented with abnormal uterine bleeding following a D&C, ultimately diagnosed with an acquired uterine AVM.

## Case report

A 34-year-old gravida 1, para 0, previously healthy woman presented at 15 weeks and 4 days gestation for a routine prenatal visit. A transvaginal ultrasound (TVUS) performed at that time revealed a fetal age of 11 weeks and 5 days, with an absence of fetal heart tones (FHT). Following a discussion with her obstetrician-gynecologist (OBGYN), the decision was made to proceed with a D&C for the missed first-trimester abortion. Postoperatively, the patient experienced an uneventful recovery, with minimal vaginal bleeding noted at her 2-week follow-up appointment.

**Fig. 1:**
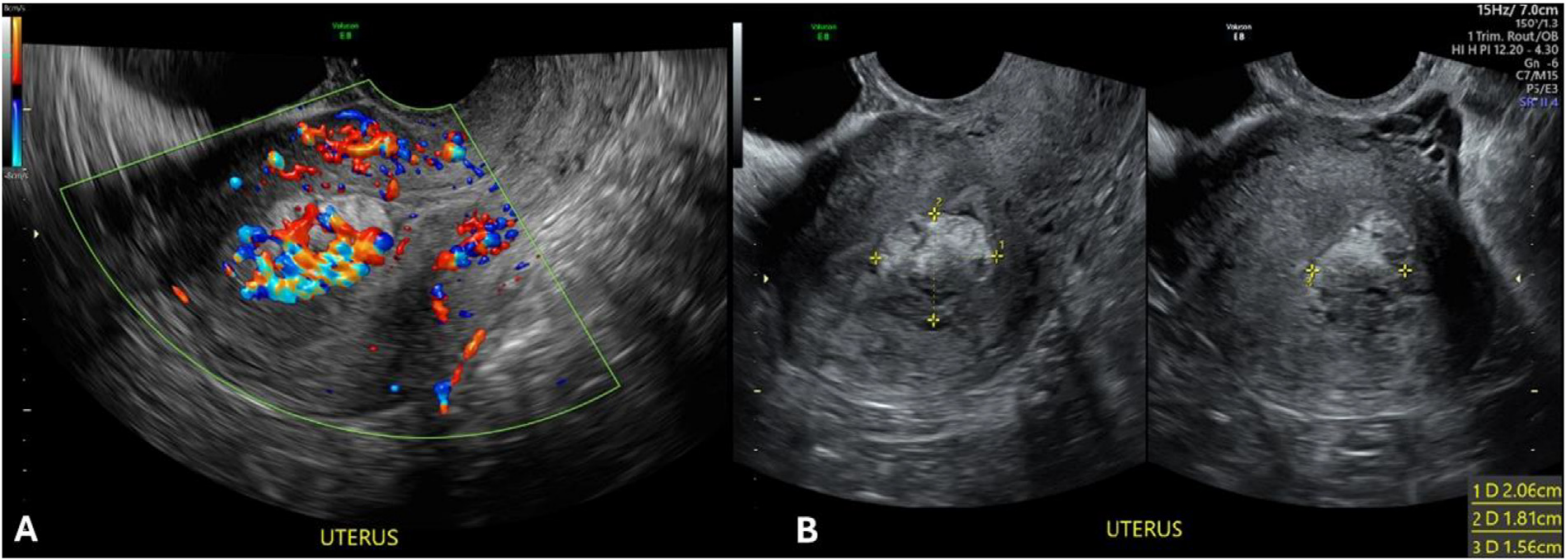
Transvaginal Ultrasound showing doppler findings (A) and spatial measurements of echogenic mass (B) Features suggestive of an increased vascular component that is indistinguishable whether it is located in the endmetrium or the myometrium.

Two months later, the patient presented with abnormal uterine bleeding. She reported a return to regular menses, followed by increased spotting, progressively severe cramping, and menorrhagia with passage of clots. Her OBGYN reviewed the benign pathology of the previously removed products of conception (POC) and performed TVUS, which showed multiple small cysts as well as an echogenic mass with increased blood flow measuring 2.1 × 1.8 × 1.6 cm (Fig. 1). Differential diagnosis included retained products of conception (RPOC) versus uterine AVM. To provide a defective diagnosis, magnetic resonance imaging (MRI) of the pelvis with contrast was then ordered on an outpatient basis. Given the patient's hemodynamic stability and absence of pain or fever, she was prescribed tranexamic acid and ferrous sulfate to manage the blood loss.

**Fig. 2:**
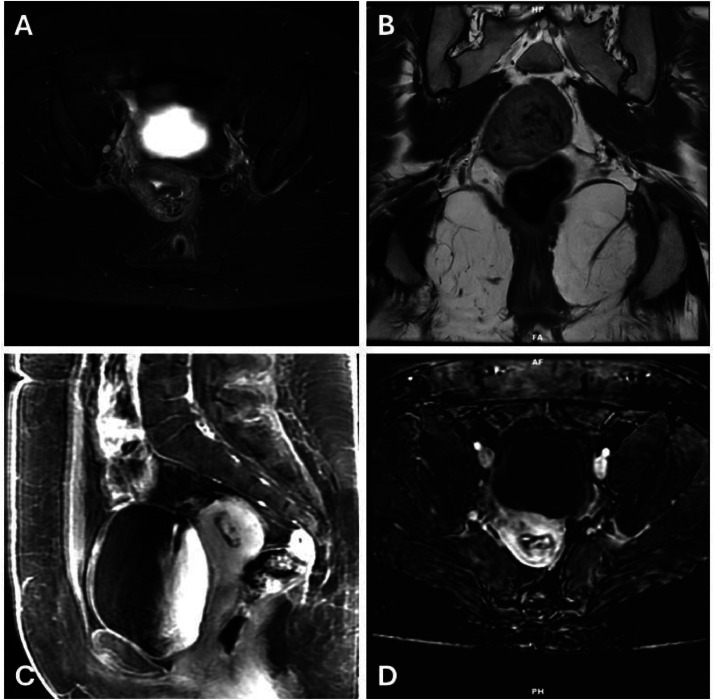
MRI – Axial Fat suppressed T2WI A), Coronal T2WI (B), Sagittal delayed postcontrast T1WI (C), and Axial Digital Subtraction Imaging (D). Features of serpiginous low T2 hypointense structures through the myometrium suggestive of myometrial vasculature pointing towards uterine AVM in concordance with hypervascular Doppler signal (A and B). Supporting this is the finding of incompletely maintained endometrialMyometrial interface on the posterior aspect (C). However, appearance of a high T1 signal mass in pre- and postcontrast images with enhancing components on subtraction imaging would favor the diagnosis of RPOC. Note: MRI, Magnetic resonance imaging; T2WI, T2-weighted imaging; T1WI, T1-weighted Imaging; AVM, Arteriovenous malformation; RPOC, Retained products of conception.

**Fig. 3:**
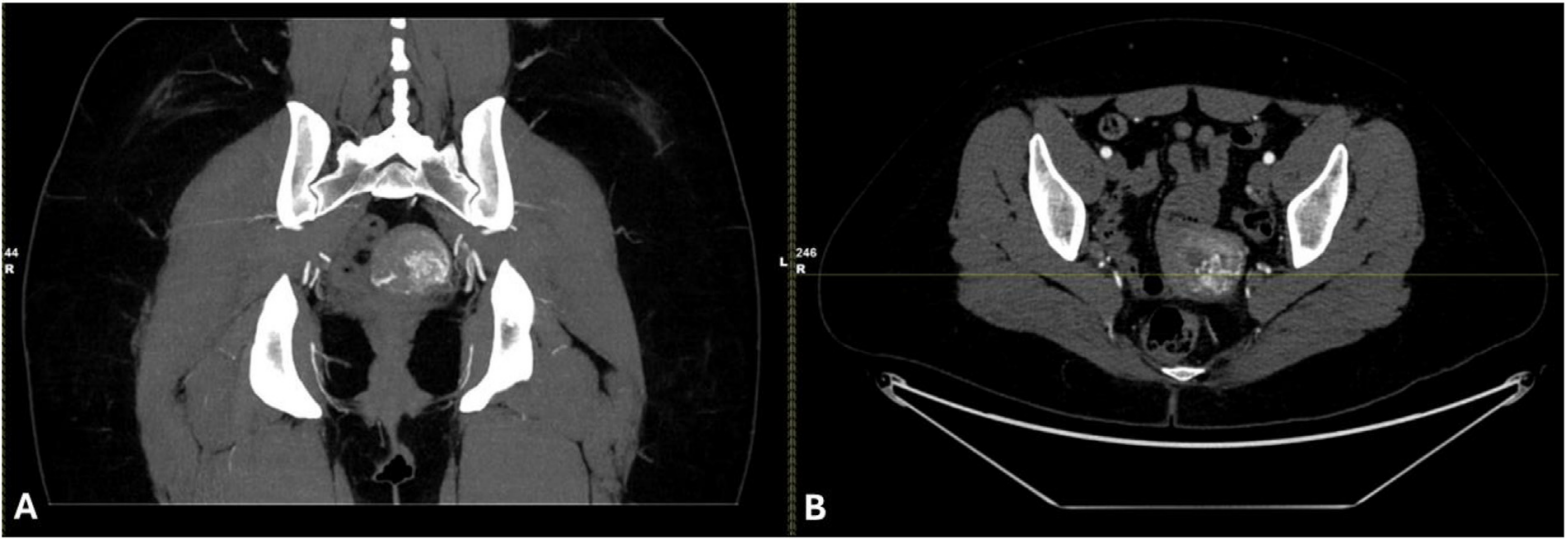
CTA in coronal (A) and axial (B) views. Clear visualization and comparisons with the prior MRI pelvis favor towards an AVM due to the early filling of the cluster of vessels that appeared to be predominantly located in the myometrium. Views also suggest that both left, and right uterine arteries are involved. Note: CTA, Computer tomography angiography; MRI, Magnetic resonance imaging; AVM, Arteriovenous malformation.

Three days after this encounter, the patient presented to the emergency department with fatigue, confusion, a presyncopal episode, abdominal pain, and vaginal bleeding. She was admitted, and an expedited MRI of the pelvis revealed a mixed signal endometrial mass with solid enhancement, an indistinct endometrial-myometrial interface in the posterior uterine body along with serpiginous low T2 signal through the myometrium - features suggestive of both RPOC (mass) and uterine AVM (dilated abnormal vessels in the myometrium; Fig. 2). Due to the indeterminate findings, an urgent computed tomography angiography (CTA) of the pelvis was performed and revealed 2.3 × 2.1 cm vessel cluster within the myometrium, supplied by both uterine arteries, with early venous filling, favoring a diagnosis of uterine AVM over RPOC (Fig. 3). The patient's symptoms had improved with conservative measures, and she opted to undergo selective left uterine artery embolization (UAE) as outpatient.

**Fig. 4:**
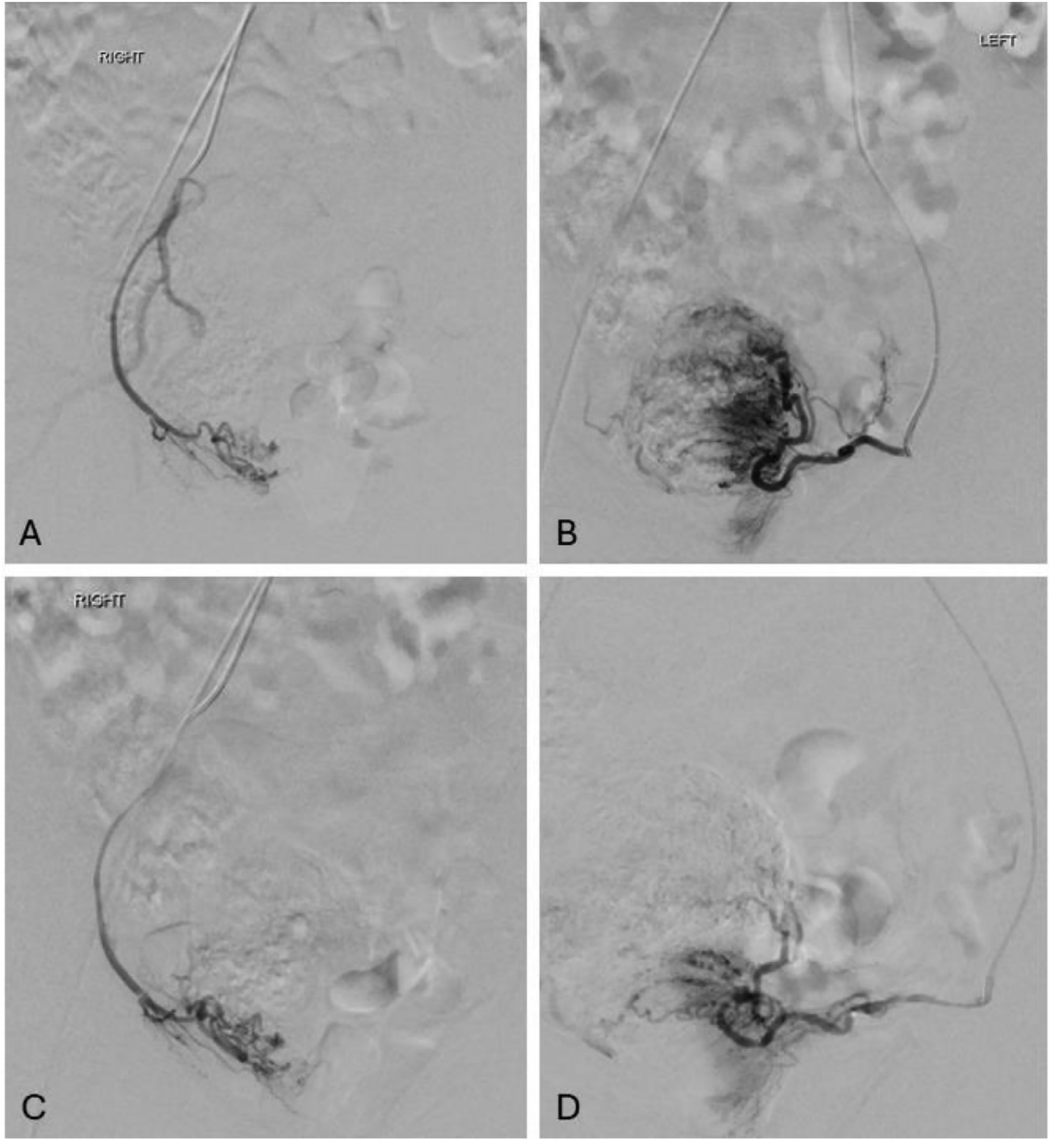
Selective angiograms of right (A and C) and left (B and D). Postembolization imaging (C and D) shows a remarkable reduction in blood flow through the AVM. Although previous imaging was able to narrow down on the diagnosis of AVM, it was mentioned that both the left and right uterine arteries likely contributed to the AVM. The above imaging clearly reveals that only the left uterine artery acted as a feeder. Embolization yielded excellent results. Note: AVM, Arteriovenous malformation.

The informed consent for the procedure was obtained and the patient was placed in the supine position. Moderate sedation with fentanyl and midazolam was used. Ultrasound-guided access to the right common femoral artery (CFA) was obtained using a 21-gauge needle, subsequently replaced by a 6 French sheath. A 5 French Roberts uterine catheter (RUC) was advanced to the left uterine artery, supplying the suspected AVM, and selective embolization with Gelfoam® (Pfizer, New York City, NY) slurry was performed, achieving stasis. Care was taken to preserve blood flow to the left ovarian artery, which originated proximally to the embolization site. Pre- and postprocedure angiograms were performed using the RUC, OmniFlush® (B. Braun Medical Inc., Bethlehem, PA) catheter, or Renegade^TM^ microcatheter (Boston Scientific, Marlborough, MA) on various arteries (Fig. 4). The left internal iliac and left uterine artery angiograms revealed hypertrophy consistent with AVM sequelae, while the right internal iliac and uterine arteries appeared normal. Postembolization left uterine artery angiograms and pelvic aortograms demonstrated appropriate reduction in arterial flow without evidence of nontarget embolization. Instruments were removed, and hemostasis of the right CFA was achieved using a Vascade® (Haemonetics, Boston, MA) vascular closure device, supplemented with 15 minutes of manual compression due to a suspected small hematoma. The skin was subsequently closed with Dermabond® (Johnson, New Brunswick, NJ). The patient was discharged within 24 hours, asymptomatic, and provided with appropriate postprocedural instructions.

**Fig. 5:**
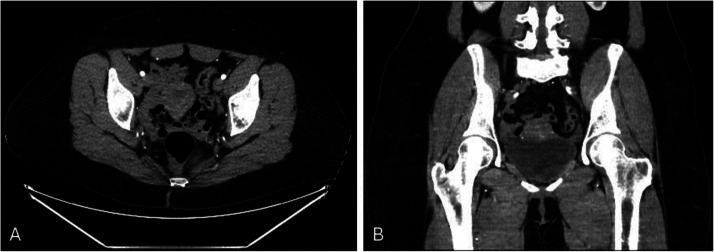
CTA images 6 weeks postembolization. As expected, when compared to CTA images shown in Fig. 3, there is no significant vascular ectasia withing the uterine parenchyma. There are no further complications such as arteriovenous fistulae or pseudoaneurysm because of the transarterial catheterization. Note: CTA, Computer tomography angiography.

Follow-up TVUS and transabdominal ultrasound 1-week postprocedure showed a decrease in the size of the previously noted echogenic thickening, with no flow consistent with the history of UAE. A 6-week follow-up CTA (Fig. 5) of the pelvis revealed no residual AVM nidus, fistula, or pseudoaneurysm, with patent bilateral internal iliac, uterine, and ovarian arteries.

## Discussion

This case illustrates the diagnostic challenges associated with acquired uterine AVMs, particularly due to the overlapping clinical presentations with other conditions such as RPOC, gestational trophoblastic disease (GTD), hemangiomas, and uterine malignancies like sarcomas [3]. Given the acute nature of uterine AVMs, prompt treatment is essential, with consideration of the patient's desire for future fertility [4]. In this case, UAE was selected as the treatment of choice, which preserved the patient's fertility. However, fertility outcomes are influenced by the anchoring and vascularity of the placenta [5].

Acquired uterine AVMs represent abnormal connections between branches of the uterine arteries and corresponding myometrial venous plexuses, typically located at the endometrial-myometrial junction and deeper within the myometrium. Although bilateral arterial supply is common, as initially suspected based on the CTA findings in this case, only a unilateral supply may be present [6]. The clinical presentation often mimics other conditions, necessitating a broad differential diagnosis. Laboratory investigations, such as beta-hCG measurement, can help exclude pregnancy-related conditions like GTD [7].

In most cases, initial imaging with TVUS or transabdominal ultrasound is recommended. However, these modalities may not adequately distinguish between AVM and RPOC, as demonstrated by several studies, including a recent review by Hamel et al. [8] Sonographic features such as hypoechoic cysts and endo- or myometrial thickening lack specificity for uterine AVMs. One reliable method for assessing the increased blood flow associated with AVMs is the measurement of peak systolic velocity (PSV) via spectral Doppler imaging, with values exceeding 40 cm/s suggesting clinically significant AVMs and increased bleeding risk. Lower values may warrant sonographic follow-up, contingent on symptoms and hemoglobin levels [9,10].

MRI with contrast is the preferred next step in diagnosis due to its superior ability to delineate vascular structures and involvement of surrounding tissues. Indeed, dynamic contrast-enhanced MRI (DCE MRI) offers higher contrast resolution than conventional CTA and provides better characterization of pelvic organs without radiation exposure [7,[10], [11], [12]]. However, CTA may be necessary in cases of patient instability, time constraints, or cost considerations. In our case, CTA was employed due to persistent ambiguity following MRI, requiring confirmation.

Further diagnostic and therapeutic options include transarterial embolization, transvenous embolization, and ultrasound-guided direct puncture. In our case, conventional noninvasive imaging was insufficient to identify the specific feeder artery responsible for the AVM. Selective angiography conclusively demonstrated the left uterine artery as the feeder vessel, which was successfully embolized. Although rare, some cases require invasive angiography for a definitive diagnosis [13,14]. In addition, there are cases where AVMs were visualized using unconventional modalities such as hysteroscopy [15]. However, the dual diagnostic and therapeutic role of invasive angiography makes it the gold standard [16,17].

Transarterial approach allows precise localization of embolization site, which is critical in younger patients desiring future fertility. Embolization distal to the origin of the ovarian artery, as performed in this case, is crucial for preserving ovarian blood supply. This ``superselective'' embolization technique has demonstrated favorable outcomes in fertility preservation [18]. Conservative management options exist for stable, nonlife-threatening uterine AVMs and are arguably the best treatment to preserve fertility. However, these options are ineffective if bleeding persists beyond 4 weeks and may require prolonged treatment [9,10]. In cases where conservative and minimally invasive approaches fail, hysterectomy may be necessary, although it carries the risk of postsurgical complications, especially in congenital uterine AVMs [10,19].

## Conclusion

In conclusion, uterine AVMs may represent a diagnostically challenging condition, as exemplified by this case, where a seemingly stable condition deteriorated rapidly. Uterine AVMs can cause significant hemorrhage with potentially catastrophic consequences. The incidence of acquired AVMs has increased, likely due to higher rates of D&C and improved imaging methods. While AVMs can be congenital, acquired AVMs are an iatrogenic reality that requires vigilant follow-up. This case underscores the importance of a multimodal diagnostic approach, utilizing TVUS, MRI, CTA, and conventional angiography, to accurately identify and manage the culprit vessel. Further research is warranted to optimize treatment strategies, particularly in preserving fertility and minimizing complications in minimally invasive and surgical interventions.

## Data availability statement

The data underlying this article will be shared on reasonable request to the corresponding author.

## Patient consent

Written informed consent for the publication of patient information and images to be published as part of the submission was provided by the patient.
